# Antimicrobial use policy change in pre-weaned dairy calves and its impact on antimicrobial resistance in commensal *Escherichia coli:* a cross sectional and ecological study

**DOI:** 10.1186/s12866-019-1576-6

**Published:** 2019-09-12

**Authors:** Josephine A. Afema, Margaret A. Davis, William M. Sischo

**Affiliations:** 1 Paul G. Allen School for Global Animal Health, College of Veterinary Medicine, Washington State University, Pullman, Washington USA; 2 Department of Pathobiology, School of Veterinary Medicine, St. George’s University, Saint George’s, Grenada; 3 Department of Veterinary Clinical Sciences, College of Veterinary Medicine, Washington State University, P. O. Box 646610, Pullman, WA 99164-7090 USA

**Keywords:** Antimicrobial use, Antimicrobial resistance, Pre-weaned dairy calves, Feedback, Policy change

## Abstract

**Background:**

This study is based on data collected to investigate the relation of peri-parturient events (colostrum quality, passive transfer of immunity, calving difficulty) on calf health and antimicrobial use. A component of the study was to provide feedback to farm management to identify calves at risk for disease and promote antimicrobial stewardship. At the start of the study (May 2016), a combination of enrofloxacin, penicillin, and sulfamethoxazole was the first treatment given to clinically abnormal calves. Based on feedback and interaction between study investigators, farm management and consulting veterinarians, a new policy was implemented to reduce antimicrobial use in calves. In August, the first treatment was changed to a combination of ampicillin and sulfamethoxazole. In September, the first treatment was reduced to only sulfamethoxazole. We investigated the effects of these policy changes in antimicrobial use on resistance in commensal *Escherichia coli*.

**Results:**

We enrolled 4301 calves at birth and documented antimicrobial use until weaning. Most calves (99.4%) received antimicrobials and 70.4% received a total of 2–4 treatments. Antimicrobial use was more intense in younger calves (≤ 28 days) relative to older calves. We isolated 544 *E. coli* from fecal samples obtained from 132 calves. We determined resistance to 12 antimicrobials and 85% of the isolates were resistant to at least 3 antimicrobial classes. We performed latent class analysis to identify underlying unique classes where isolates shared resistance patterns and selected a solution with 4 classes. The least resistant class had isolates that were mainly resistant to only tetracycline and sulfisoxazole. The other 3 classes comprised isolates with resistance to ampicillin, chloramphenicol, aminoglycosides, sulfonamides, tetracycline, in addition to either ceftiofur; or nalidixic acid; or ciprofloxacin plus nalidixic acid and ceftiofur. Overall, *E coli* from younger calves and calves that received multiple treatments were more likely to have extensive resistance including resistance to fluoroquinolones and ceftiofur. In general, there was a declining trend in resistance to most antimicrobials during and after policy changes were implemented, except for ampicillin, ciprofloxacin, ceftiofur and gentamicin.

**Conclusions:**

Information feedback to farms can influence farm managers to reduce antimicrobial use and this can change endemic farm resistance patterns.

**Electronic supplementary material:**

The online version of this article (10.1186/s12866-019-1576-6) contains supplementary material, which is available to authorized users.

## Background

Antimicrobials are used in pre-weaned calves to prevent infections and to treat diseases such as diarrhea and pneumonia [1]. Antimicrobial use in animals and humans is known to select for resistant bacteria and interventions to reduce antimicrobial use have been shown to decrease prevalence of resistant bacteria or result in their disappearance [2, 3]. However, some resistant bacterial clones or resistance determinants may persist after antimicrobials are discontinued, highlighting the complex interactions that exist between antimicrobial use and resistance [2, 4].

Several observational studies have described the relationship between antimicrobial use in animal production and resistance using *Escherichia coli* as the indicator species [5–7]. *Escherichia coli* is an important indicator organism for monitoring antimicrobial resistance in healthy food animals. Antimicrobial use in food animals exerts selection pressure on gut microbiota and *E. coli* is a good indicator of antimicrobial use and a potential reservoir of resistance determinants for bacteria of public health significance. *Escherichia coli* is a common commensal in the gastrointestinal tract of food animals, it is easy to culture and isolate from stool, and to perform antimicrobial resistance testing [8, 9].

Other studies have quantified the overall amounts of antimicrobials sold or consumed at farm or national levels and determined associations with resistance [6, 10]. While such studies provide pertinent information on the overall impact of antimicrobial consumption on resistance in different animal production sectors, they are limited at providing information on intricate interactions that occur between antimicrobial use and resistance at the individual animal or herd level [11]. Studies that document farm-specific antimicrobial use and resistance provide deeper insights into the relationship between antimicrobial use and resistance at the animal level [12].

Different approaches are used to evaluate antimicrobial consumption in animals and humans. Antimicrobial use is often assessed in “technical units” per population at risk for a given period. Technical units can be treatment costs, number of packages used, weight of active antimicrobial substance, or other indicators. The number of individuals treated or the number of prescriptions per 1000 people per day has been proposed for measuring antimicrobial consumption. This approach accounts for exposure of individuals to antimicrobials and it is suitable for studying the association between antimicrobial use and resistance [10, 13].

Analyzing trends in antimicrobial resistance is useful in assessing whether measures taken to reduce antimicrobial consumption result in reduced resistance. For instance, decreasing temporal trends in antimicrobial resistance in commensal *E. coli* from livestock in Belgium between 2011 and 2014 was attributed to reduction in overall consumption of veterinary antimicrobials during 2011–2013 [14]. Policies aimed at reducing antimicrobial use in livestock in the Netherlands resulted in decreased resistance in *E. coli* from swine and veal but a clear association was not seen in dairy cattle [15]. There is therefore need for studies in dairy cattle to better understand association between antimicrobial use and resistance.

The goal of this study was to assess the effect of antimicrobial use policy changes implemented to reduce the amounts of antimicrobials used in pre-weaned calves on resistance using commensal *E. coli* as indicator species.

## Results

### Antimicrobial use

From May 2016 – January 2017, data on antimicrobial use was obtained from a total of 4301 calves. Over this period, the antimicrobial use policies developed by farm management and the use of antimicrobials by the care team changed and impacted the amounts of antimicrobials being used to treat pre-weaned calves. There were three distinct time periods associated with these changes: May – August 2016 (pre-policy change), September 2016 – mid-November 2016 (implementation of the policy change), and mid-November 2016 – Jan 2017 (post policy change). During these three-time periods; 1236, 1722 and 1344 calves respectively, were monitored for antimicrobial use. Regardless of the time period, nearly all of the 4301 calves (99.4%) received antimicrobials by the time of weaning. Over the study period, nearly all calves were treated with sulfamethoxazole (97.8%), approximately 75% received ampicillin treatment, and 55% of the calves received enrofloxacin treatment. Fewer than 16% of calves received florfenicol (15.6%), tulathromycin (11.8%), or ceftiofur (3.3%) treatment.

Antimicrobial treatment was defined as the administration of a full dose of a single antimicrobial or a combination of two or more antimicrobials for a given condition. A summary of the antimicrobial treatments that were administered is presented in Additional file 1: Table S1. In addition, we evaluated the cumulative number of antimicrobial treatments from birth to weaning and 70.4% of the calves received a total of 2–4 treatments by weaning time (Table 1).

**Table 1:** Cumulative number of antimicrobial treatments administered to calves (*n* = 4301) from birth to weaning

No. treatments	No. of calves	%
0	26	0.6
1	534	12.4
2	1119	26.0
3	1206	28.0
4	705	16.4
5	368	8.6
6	172	4.0
7	69	1.6
8	37	0.9
9	26	0.6
10–17	37	0.9

**Fig. 1:**
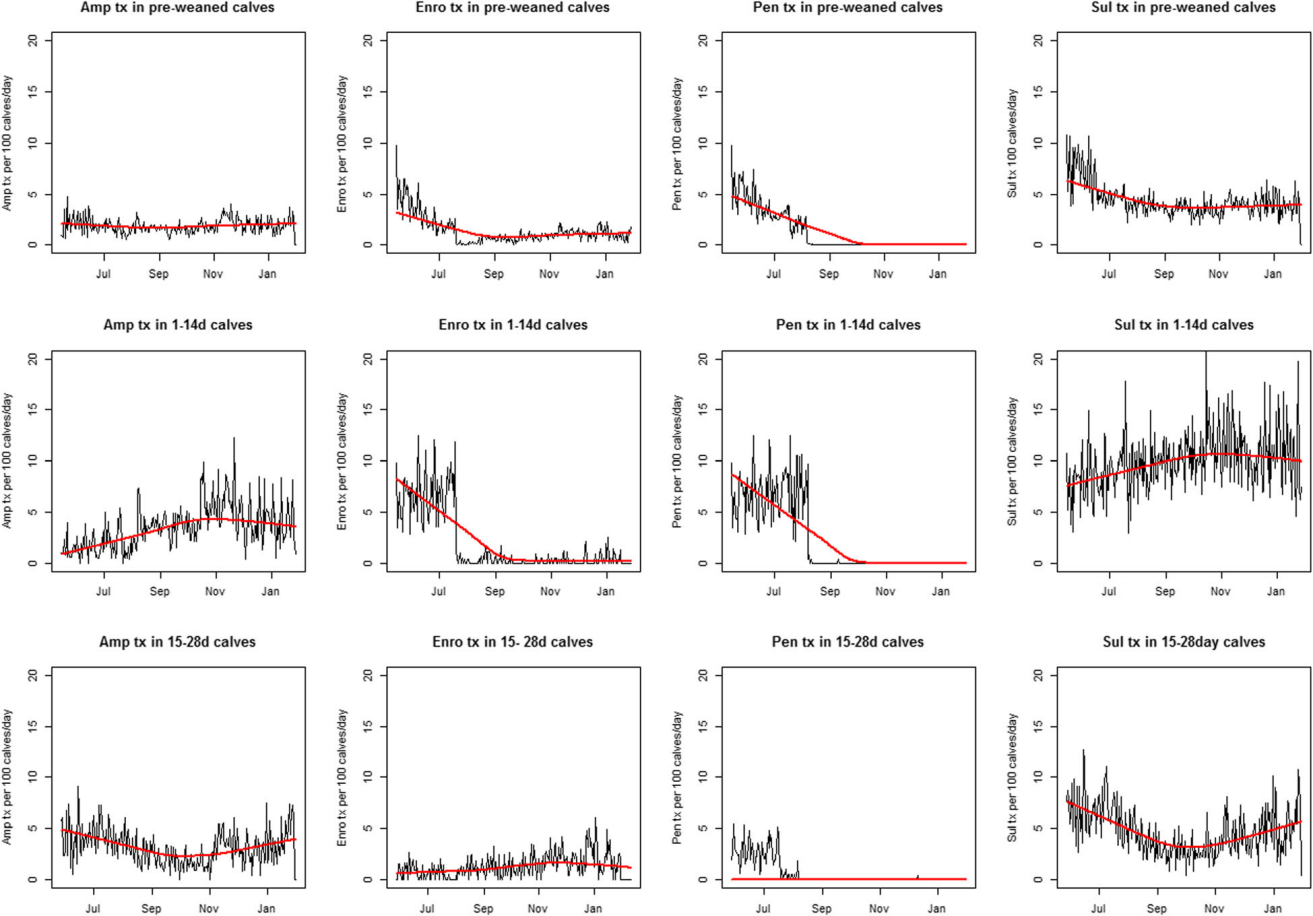
Treatment intensity for ampicillin, enrofloxacin, penicillin and sulfonamide in pre-weaned calves stratified by age

Marked changes in treatment intensity were associated with the change in antimicrobial use policy following monitoring and outreach to the farm management. Treatment intensity for the four most frequently used antimicrobials was assessed as the number of calves treated with an antimicrobial per day per 100 calves by age (Fig. 1). When all ages were considered (birth through weaning), ampicillin use was fairly constant during the study period, whereas intensity of enrofloxacin and sulfonamide use decreased during the implementation of the policy change and remained fairly constant through the post policy change time period. Specifically, in 1 – 14d old calves, ampicillin and sulfonamide use increased during implementation of the policy change and remained constant through the post implementation interval. Enrofloxacin use decreased to near zero during implementation of the policy change and remained low while penicillin use was discontinued as part of the policy change. In 15 – 28d old calves, ampicillin use was fairly consistent and there was a low but consistent use of enrofloxacin across all three-time periods.

**Fig. 2:**
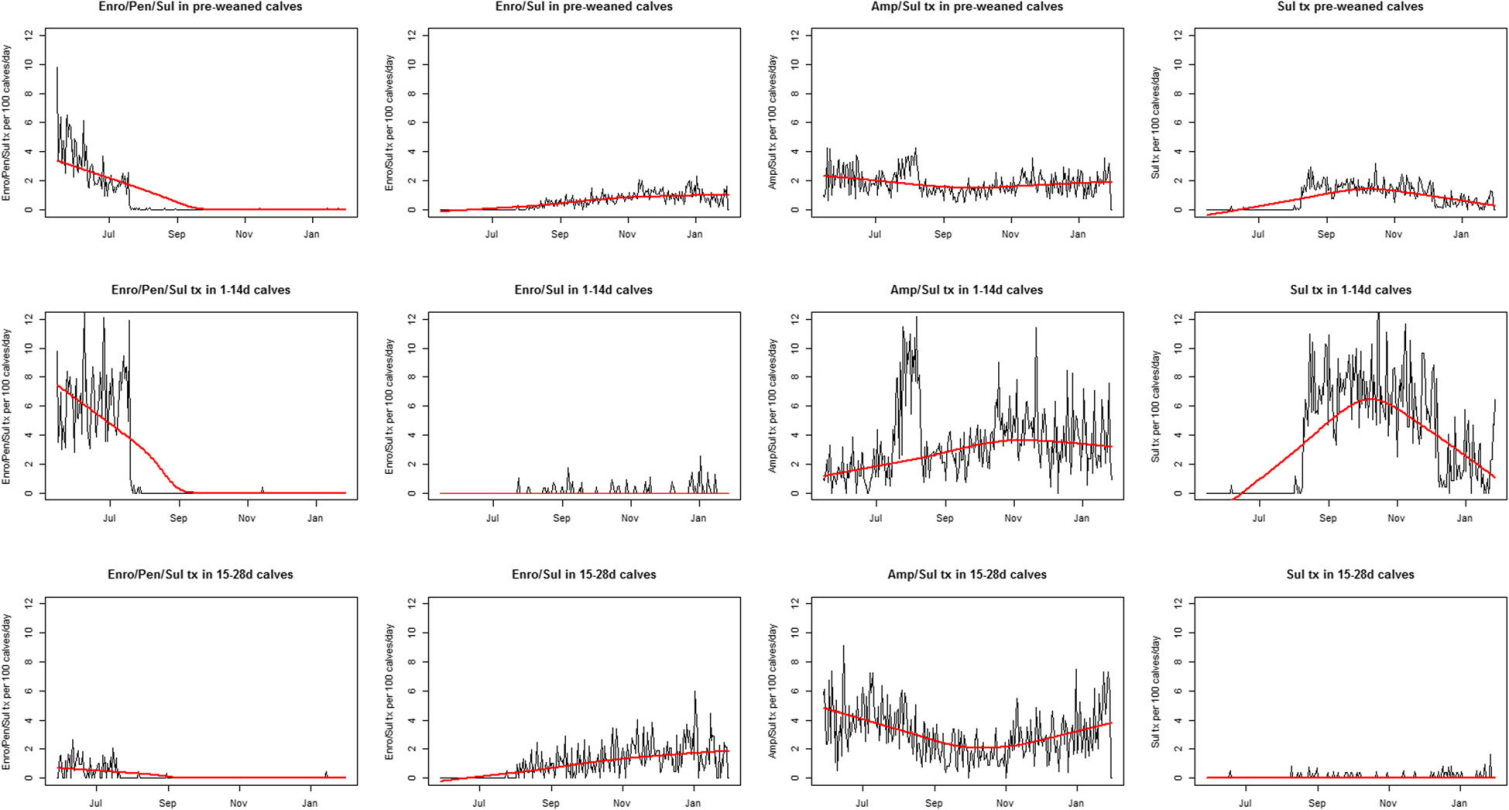
Treatment intensity for combined or single antimicrobial treatments in pre-weaned calves stratified by age

Treatment intensity for combined and single antimicrobial treatments varied among age groups and changed over time (Fig. 2). Before the policy change, the first treatment administered was a combination of parenteral enrofloxacin, parenteral penicillin and oral sulfonamide used as a single treatment (separate syringes for parenteral administration but administered simultaneously) in 1 – 14d old calves. This treatment targeted calves with diarrhea or assessed as abnormal because of inappetance or depressed attitude or perception of risk for an unhealthy state. This combination treatment was discontinued with the implementation of the antibiotic use policy change in August and replaced with an oral sulfonamide as first treatment (Table 2). During and after the implementation of the policy change, a combination of enrofloxacin and sulfonamide was used as a third treatment and predominantly in 15 – 28d old calves. In all age groups, the intensity of ampicillin and sulfamethoxazole use showed a decreasing trend with a trough around October and increased slightly until January. Overall, there was a modest increasing trend in ampicillin/sulfamethoxazole use in 1 – 14d old calves through November then leveled off until January. In 15 – 28d old calves, treatment intensity of ampicillin/sulfamethoxazole decreased from the start of the study, reached a trough around October, and then increased slightly for the rest of the study. While the overall treatment intensity of antimicrobial use changed as consequence of the policy change, (i.e the quantity and numbers of antimicrobials administered), the pattern of use did not change across the policy change as median days for first, second and third treatments were consistent between the study periods (Table 2).

**Table 2:** Median time to first, second and third treatment stratified by time periods associated with change in antimicrobial use policy

Period	Treatment	Median	Interquartile range	Predominant drugs used
Pre-policy change				
	First	9 days	6–12 days	Enro/Pen/Sul combination
	Second	17 days	13–22 days	Amp/Sul combination
	Third	25 days	20–36 days	Amp/Sul/Mac combination
Policy change				
	First	8 days	7–9 days	Sul
	Second	14 days	12–19 days	Amp/Sul combination
	Third	32 days	21–48 days	Enro/Sul or Nuf/Sul combination
Post change				
	First	8 days	6–10 days	Sul, Sul/Spc combination
	Second	13 days	11–16 days	Amp/Sul combination
	Third	21 days	16–31 days	Enro/Sul or Amp/Sul combination

Enro: enrofloxacin; Pen: penicillin; Sul: sulfonamide; Mac: macrolide; Spc: spectinomycin; Nuf: florfenicol

Of note and not included in the general farm policy was that calves between May until end of July were given prophylactic tylosin usually during the first 3 days post-calving, and oxytetracycline in milk replacer from day 22 until weaning, but these drugs were discontinued in August. Neither of these treatments were recorded by calf care workers.

### Antimicrobial resistance in commensal *E. coli* from pre-weaned calves

From May 2016 – January 2017, we collected 140 fecal samples from 132 calves (8 calves were sampled twice) over 14 sampling occasions spaced at biweekly intervals. Relative to the intervals associated with the policy change, 30, 50, and 60 fecal samples were collected prior to the policy change, during the policy change, and after the policy change, respectively. At each sampling time, a fecal sample was collected from a calf in a weekly age category (week 1 of age to week 10 of age) to reflect the pre-weaning period. We obtained a total of 544 *E. coli* isolates (average of 4 isolates/fecal sample) and determined susceptibility to 12 antimicrobials. A small percentage of isolates were pan-susceptible or susceptible to all tested antimicrobials (5.3%) or resistant to a single or only two antimicrobials (4.4 and 5.1%, respectively). The majority of the isolates (85%) were resistant to at least 3 antimicrobial classes. A high percentage of isolates (> 65%) were resistant to tetracycline, sulfisoxazole, chloramphenicol, kanamycin, streptomycin, and trimethoprim/sulfamethoxazole (Table 3). A smaller proportion of isolates (25–50%) were resistant to nalidixic acid, gentamicin, ceftiofur and ciprofloxacin, and only 3% of isolates were resistant to amikacin.

**Table 3:** The percentage of isolates resistant to each of 12 antimicrobials (*n* = 544)

Antimicrobial	No. resistant isolates	%
Tetracycline	501	92.1
Sulfisoxazole	468	86.0
Chloramphenicol	406	74.6
Kanamycin	402	73.9
Streptomycin	400	73.5
Trimethoprim/sulfamethoxazole	372	68.4
Ampicillin	322	59.2
Nalidixic acid	260	47.8
Gentamicin	175	32.2
Ceftiofur	148	27.2
Ciprofloxacin	138	25.4
Amikacin	15	2.8

**Fig. 3:**
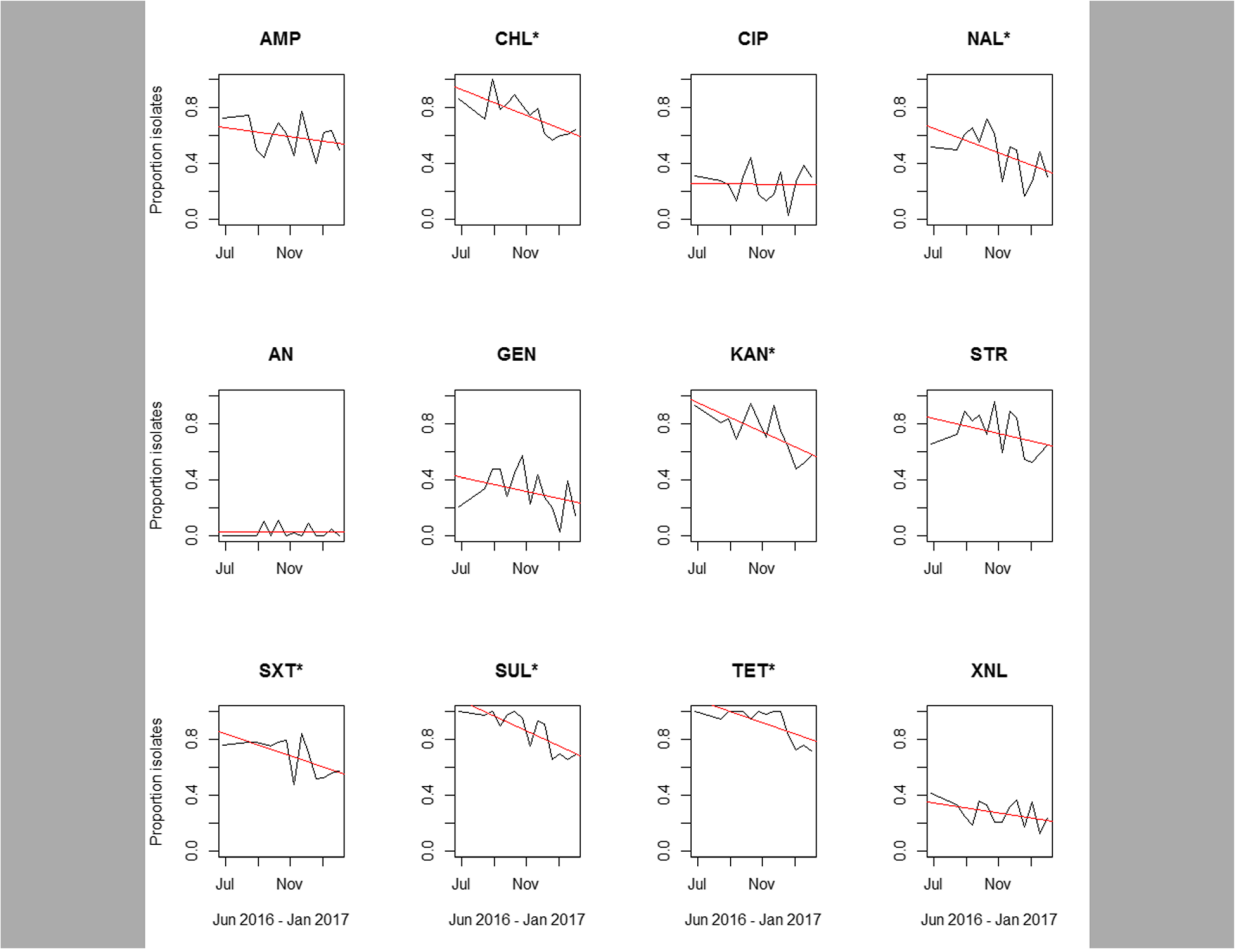
Trends in the proportion of isolates that are resistant to 12 antimicrobials over time. AMP: ampicillin; CHL: chloramphenicol; CIP: ciprofloxacin; NAL: nalidixic acid; AN: amikacin; GEN: gentamicin; KAN: kanamycin; STR: streptomycin; SXT: trimethoprim/sulfamethoxazole; SUL: sulfisoxazole; TET: tetracycline; XNL: ceftiofur. ^*^Trend is statistically significant

The proportion of resistant isolates to individual antimicrobials was plotted over time and trends in resistance was evaluated using simple linear regression analysis (Fig. 3). While there was a time dependent decreasing trend in resistance proportion for all antimicrobials except for amikacin which stayed near zero and ciprofloxacin (25% resistant), these trends were not statistically significant for ampicillin, ceftiofur, and streptomycin.

### Antimicrobial resistance structure

Antimicrobial resistance data for the 544 isolates was analyzed using LCA to identify classes of isolates with shared resistance patterns. Amikacin was excluded from the analysis because 97% of isolates were susceptible. The analysis was started by fitting a model with 3 classes, followed by running models with 4, 5, and 6 classes. A model with 4 classes was selected because it was parsimonious, provided meaningful interpretation, and fit the data well according to Akaike information criteria and Bayesian information criteria (Additional file 1: Table S2). The proportion of isolates in each class, and the probability of resistance of isolates in each class to the 11 antimicrobials is shown in Table 4.

**Table 4:** Latent classes of antimicrobial resistance in *E. coli* (n = 544) from pre-weaned dairy calves

Antimicrobial resistance class	TET	XNL^+^	NAL^+^	CIP + NAL + XNL^+^
Latent class prevalence	30.8%	19.9%	26.1%	23.5%
Item response probabilities				
Ampicillin	0.146	**0.969**	0.487	**1.000**
Chloramphenicol	0.420	**0.770**	**0.992**	**0.915**
Ciprofloxacin	0.006	0.000	0.066	**0.982**
Nalidixic acid	0.020	0.036	**0.950**	**0.991**
Gentamicin	0.000	0.243	**0.535**	**0.633**
Kanamycin	0.282	**0.960**	**0.955**	**0.969**
Streptomycin	0.279	**0.888**	**0.978**	**0.966**
Sulfisoxazole	**0.587**	**1.000**	**1.000**	**1.000**
Trimethoprim/sulfamethoxazole	0.082	**0.915**	**0.969**	**1.000**
Tetracycline	**0.800**	**1.000**	**1.000**	**0.992**
Ceftiofur	0.007	**0.778**	0.000	**0.503**

Item response probabilities of > 0.5 are highlighted in bold

The largest class comprised 30.8% of the isolates and these isolates had a high probability of resistance to tetracycline and moderate resistance to sulfisoxazole and this class was named TET. The other 3 classes comprised isolates with moderate to high probability of resistance to ampicillin, chloramphenicol, kanamycin, streptomycin, sulfonamides, tetracycline, in addition to either ceftiofur, or nalidixic acid, or ciprofloxacin plus NAL and XNL. These classes are therefore referred to as XNL^+^, NAL^+^, and CIP + NAL + XNL^+^ respectively (Table 4).

### Multinomial logistic regression analysis

To determine the effects of shifts in antimicrobial use (exposure) on resistance (outcome), we performed cross tabulations and ran univariable models showing associations between each explanatory variable and resistance class (Table 5). There was association between antimicrobial treatment and classes with extensive resistance. Isolates from untreated calves compared to calves treated once were less likely to be in a class with extensive resistance. Furthermore, isolates from calves treated multiple times were more likely than calves treated once to be in a class with extensive resistance compared to the TET class.

**Table 5:** Univariable models of associations between antimicrobials, number of treatments, age, time and antimicrobial resistance LCA classes for 546 commensal *E. coli* isolated from fecal samples obtained from 140 calves

Risk factors	Cross tabulations				Resistance classes (odds ratio & 95% CI)		
	TET	XNL^+^	NAL^+^	CIP + NAL + XNL^+^	XNL^+^	NAL^+^	CIP + NAL + XNL^+^
Enrofloxacin							
Untreated	123	95	71	69			
Treated	51	19	52	64	**0.5 (0.3–0.9)**	**1.8 (1.1–2.9)**	**2.2 (1.4–3.6)**
Sulfonamide							
Untreated	28	34	4	7			
Treated	146	80	119	126	**0.5 (0.3–0.8)**	**5.7 (1.9–16.7)**	**3.5 (1.5–8.2)**
β-lactams							
Untreated	71	56	24	40			
Treated	103	58	99	93	0.7 (0.4–1.4)	**2.8 (1.7–4.9)**	**1.6 (1.0–2.6)**
No. of treatments							
×1	56	35	15	35			
0	23	31	4	7	**2.2 (1.1–4.3)**	0.6 (0.2–2.2)	0.5 (0.2–1.3)
×2	58	20	41	34	0.6 (0.3–1.1)	**2.6 (1.3–5.3)**	0.9 (0.5–1.7)
×3	24	19	47	36	1.3 (0.6–2.6)	**7.3 (3.4–15.5)**	**2.4 (1.2–4.7)**
×4–6	13	9	16	21	1.1 (0.4–2.9)	**4.6 (1.8–11.6)**	**2.6 (1.1–5.8)**
Age (5 categories)							
57 - 80d	63	14	17	14			
43 - 56d	44	16	36	10	1.6 (0.7–3.7)	**3.0 (1.5–6.1)**	1.0 (0.1–0.4)
29 - 42d	28	11	52	20	1.8 (0.7–4.4)	**6.9 (3.4–13.9)**	**3.2 (1.4–7.3)**
15 - 28d	15	21	14	57	**6.3 (2.6–15.2)**	**3.5 (1.4–8.5)**	**17.1 (7.6–38.5)**
1 - 14d	24	52	4	32	**9.7 (4.6–20.7)**	0.6 (0.2–2.0)	**6.0 (2.7–13.1)**
Age (2 categories)							
29 - 80d	135	41	105	44			
1 - 28d	39	73	18	89	**6.2 (3.7–10.4)**	0.6 (0.3–1.1)	**7.0 (4.2–11.6)**
Sampling period							
Nov 15 – Jan	97	54	30	64			
Sep – Nov 15	55	34	67	42	1.1 (0.6–1.9)	**3.9 (2.3–6.8)**	1.2 (0.7–1.9)
May – Aug	22	26	26	27	**2.1 (1.1–4.1)**	**3.8 (1.9–7.7)**	**1.9 (1.0–3.5)**

OR and 95% confidence intervals that exclude the null value of 1 are highlighted in bold

Study period was categorized into 3 phases: June – August, pre-antimicrobial policy change; September – mid-November, era of antimicrobial policy change; and mid-November – January, post-antimicrobial policy change. Overall, isolates obtained before the policy change were more likely than isolates collected after the policy change to be in a class with extensive resistance compared to the TET class.

We evaluated whether age was a potential confounder in the association between antimicrobial treatment and resistance and we found that young calves (≤ 28d) had higher risk for extensive resistance and age was associated with antimicrobial treatment. In addition, we evaluated whether time was an effect modifier in the association between antimicrobial treatment and resistance since antimicrobial treatments changed with time. Effect modification was present, so we stratified the data by time and determined stratum specific estimates of the effects of risk factors on resistance.

Before the policy changes were implemented (Jun-Aug), isolates from 1 – 14d old calves compared to oldest calves were most likely to be in the XNL^+^ and CIP + NAL + XNL^+^ resistance classes than the TET class (Table 6). Similarly, in the transition period, isolates from 1 – 14d old calves were most likely to be in the XNL^+^ and CIP + NAL + XNL^+^ classes. However, in the post-policy change period, isolates from 1 – 14d old calves were not associated with the CIP + NAL + XNL^+^ class. Instead, isolates from 15 – 28d old calves had the greatest likelihood to be in the CIP + NAL + XNL^+^ class. This change is also visualized in the cross tabulations. Also, isolates from 29 – 42d old calves were associated with all the extensive resistance classes (NAL^+^, XNL^+^ and CIP + NAL + XNL^+^) before policy changes were implemented, but there was no association with extensive resistance in the transition period, and in the post policy change period, an association with the NAL^+^ class reappears (Table 6).

**Table 6:** Univariable logistic regression analysis of the association between age and resistance class of commensal *E. coli* stratified by policy implementation interval

Period	Age	Cross tabulations				Odds ratio and (95% CI)		
		TET	XNL^+^	NAL^+^	CIP+NAL+XNL^+^	XNL^+^	NAL^+^	CIP+NAL+XNL^+^
Pre Policy Implementation (May-Aug)	57 - 80d	10	1	3	0			
	43 - 56d	5	4	8	1	**8 (2.4 – 26.6)**	**5.3 (2.2 – 13.0)**	2 (0.4 – 8.9)
	29 - 42d	1	4	13	5	**40 (8.9 - 179)**	**43.3 (13 – 143.8)**	**50 (4.1 – 609.1)**
	15 - 28d	4	5	2	9	**12.5 (3.8 – 41.5)**	1.7 (0.6– 5.7)	**22.5 (6.8 – 74.7)**
	1 - 14d	2	12	0	12	**60 (16.4 – 220.6)**	1.7 (0.4 – 6.9)	**60 (16.4 – 220.6)**
During Policy Implementation (Sep-Nov 15)	57 - 80d	18	2	12	4			
	43 - 56d	13	1	23	3	0.7 (0.2 – 2.6)	**2.7 (1.6 – 4.5)**	1 (0.4 – 2.2)
	29 - 42d	15	4	20	5	**2.4 (1.0 – 5.9)**	**2 (1.2 – 3.3)**	1.5 (0.7 – 3.3)
	15 - 28d	7	8	12	12	**10.3 (4.2 – 25.3)**	**2.6 (1.4 – 4.7)**	**7.7 (3.8 – 15)**
	1 - 14d	2	19	0	18	**85.5 (28.5 – 256.9)**	0.8 (0.2 – 2.9)	**40.5 (16.5 – 99.6)**
Post Policy Implementation (Nov 15-Jan)	57 - 80d	35	11	2	10			
	43 - 56d	26	11	5	6	1.3 (0.8 – 2.1)	**3.4 (1.4 – 8.4)**	0.8 (0.4 – 1.5)
	29 - 42d	12	3	19	10	0.8 (0.4 – 1.6)	**27.7 (12.4 – 61.6)**	**2.9 (1.8 – 4.8)**
	15 - 28d	4	8	0	36	**6.4 (3.2 – 12.9)**	**4.4 (1.2 – 16.1)**	**31.5 (17.3 – 57.4)**
	1 - 14d	20	21	4	2	**3.3 (2.0 – 5.4)**	**3.5 (1.4 – 8.6)**	**0.4 (0.2 – 0.9)**

OR and 95% confidence intervals that exclude the null value of 1 are highlighted in bold.

Multivariable models with antimicrobial treatment (β-lactam, enrofloxacin, and sulfonamide) as exposure variables, and age as a confounder were run for the different time periods. To simplify the analysis, age was categorized as a binary variable. We provide results for the transition and post-policy change periods (Table 7) and not for the pre-policy change period because the number of isolates relative to the number of variables evaluated was inadequate to provide meaningful results. In the transition and post-policy change periods, enrofloxacin treatment was associated with the most extensive class (CIP + NAL + XNL^+^), however, an association between enrofloxacin treatment and the NAL^+^ class was only observed in the transition period but not the post-policy change period. Furthermore, sulfonamide treatment was associated with extensive resistance only during the post-policy change period.

**Table 7:** Multivariable models of the associations between antibiotic treatments, age and resistance class stratified by time

Time	Risk factors	Cross tabulations				Resistance classes (odds ratio & 95% CI)		
		TET	XNL^+^	NAL^+^	CIP + NAL + XNL^+^	XNL^+^	NAL^+^	CIP + NAL + XNL^+^
Post	Enrofloxacin							
	Untreated	70	42	21	22			
	Treated	27	12	9	42	0.9 (0.4–2.2)	0.8 (0.3–2.2)	**6.4 (2.4–16.9)**
	Sulfonamide							
	Untreated	25	9	4	2			
	Treated	72	45	26	62	**6.3 (1.9–20.5)**	0.7 (0.1–4.8)	**22.7 (4.0–127.3)**
	β-lactams							
	Untreated	43	23	5	13			
	Treated	54	31	25	51	2.3 (0.8–6.0)	**4.8 (1.2–19.5)**	**3.3 (1.1–9.8)**
	Age							
	29 - 80d	73	25	26	26			
	1 - 28d	24	29	4	38	**13.9 (4.5–43.3)**	0.9 (0.2–4.8)	**48.1 (14–165.3)**
Transition	Enrofloxacin							
	Untreated	46	32	38	34			
	Treated	9	2	29	8	1.6 (0.3–9.7)	**4.4 (1.7–11.1)**	**7.2 (1.7–30.1)**
	Sulfonamide							
	Untreated	1	13	0	5			
	Treated	54	21	67	37	0.1 (0.01–1.2)	NA	0.5 (0.05–5.2)
	β-lactams							
	Untreated	22	23	19	19			
	Treated	33	11	48	23	1.2 (0.4–3.8)	1.2 (0.4–3.8)	2.0 (0.6–4.7)
	Age							
	29 - 80d	46	7	55	12			
	1 - 28d	9	27	12	30	**13.4 (3.7–49.1)**	2.2 (0.8–6.2)	**31.8 (8.4–120.3)**

*NA* not applicable because cell count is 0

OR and 95% confidence intervals that exclude the null value of 1 are highlighted in bold

## Discussion

The results reported here are part of an on-farm project that investigated peri-parturient events and practices that affect calf health and gave feedback to farm management on critical calf management processes including antimicrobials used. The goal of the project was to improve calf care and promote antimicrobial stewardship. The feedback and interaction between study investigators, farm management, and consulting veterinarians resulted in important changes in antimicrobial use policy that reduced the combinations and amounts of antimicrobials used to treat calves identified by workers as unhealthy. These policy changes provided an opportunity to investigate their effects on resistance in commensal *E. coli* as an indicator species.

In general, the likelihood of extensive resistance was higher before antimicrobial use policy changes were implemented compared to the period thereafter. In addition, a significant decline in resistance trends to most antimicrobials was observed after policy changes were implemented. These findings suggest that policies aimed at reducing the amounts and types of antimicrobials used in calves can result in a downward trajectory in resistance levels to some antimicrobials. Other studies have observed monotonic and/or non-monotonic relationships between antimicrobial use and resistance where reduced use was associated with either decreased, increased, or no change in resistance [15, 16].

It is important to note that the policy changes associated with the decline in observed resistance occurred following careful monitoring and summarizing antimicrobial use per animal. While there were on farm record systems designed to capture antimicrobial use, the policy change was unlikely to occur without active feedback and consultation with farm management and their veterinary consultants. Another important point is that this farm had treatment protocols and a set of antimicrobials to support the protocols, but the day-to-day work was not closely monitored and not reflected in on-farm records and calf care workers introduced combinations of approved antimicrobials into their treatment routines which were not on protocols. This underscores the importance of feedback and education backed up by real-time data and validation in creating and fostering judicious use of antimicrobials.

The antimicrobial use data is unique in that it was collected over a period of 9 months and represents a sizeable number of calves (*n* = 4301) observed from birth until weaning. We summarized antimicrobial consumption using the number of calves treated per 100 calves per day. This approach has been proposed for studying the association between antimicrobial use and resistance [10, 13]. To our knowledge, this approach has not been used in cattle previously and could be valuable in future. Data on antimicrobial use and samples for resistance were concurrently collected and this allowed for assessment of temporal relationships between antimicrobial use and resistance.

Only approximately 15% of the isolates had no or low-level resistance, whereas the majority of the isolates had extensive resistance to 8–11 antimicrobials. The practice of combined antimicrobial therapy and multiple treatments most likely selected for multiple resistance traits and this could explain the extensive resistance observed in a high percentage of isolates especially in the pre-policy change isolates [1, 17]. This is supported by our univariable model showing time-dependent reductions in multi-drug resistance and multivariable model showing time-dependent reductions in NAL^+^ class. Another explanation could be that resistance to “older antimicrobials” such as ampicillin, streptomycin, sulfonamides, and tetracycline is common in livestock even in the absence of antimicrobial selection pressure. Commensal *E. coli* with multi-resistance to aminoglycosides, tetracycline, and sulfisoxazole are widely distributed and maintained in animal production systems [8, 16]. Other studies have also reported that resistance to “older antimicrobials” is common in commensal *E. coli* from pre-weaned calves [15, 18].

A notable finding of this study is that antimicrobial resistance patterns changed in the different age groups with time. These results are most likely due to changes in antimicrobial use in the different age groups. For instance, before and during the policy change, isolates from 1 – 14d old calves were highly associated with extensive resistance, but this was not the case after the policy change. This suggests that reductions in the amounts and types of antimicrobials used in this age group resulted in reduced resistance. Conversely, isolates from 15 – 28d old calves were 22.5 times more likely to be in the CIP + NAL + XNL^+^ resistance class before the policy change. The likelihood decreased to 7.7 during the policy change but increased to 31.5 after the policy change. Ampicillin and enrofloxacin were fairly consistently used in 15 – 28d old calves during the study period; hence persistence of the most resistant class could be explained by antimicrobial selection pressure.

Reduced enrofloxacin use was one of the most significant farm’s use policy changes that occurred. The linear regression models showed a time-dependent decrease in the prevalence of nalidixic acid resistance which was not observed for ciprofloxacin resistance. Similarly, the multivariable model indicated the NAL^+^ class waned but the most resistant class (CIP + NAL + XNL^+^) persisted after the policy change.

Enrofloxacin treatment was associated with the NAL^+^ and CIP + NAL + XNL^+^ classes in the transition period, and with the CIP + NAL + XNL^+^ class only in the post policy change period. Commensal *E. coli* from pre-weaned calves treated with enrofloxacin have been reported to be resistant to ciprofloxacin and third generation cephalosporin (ceftriaxone) [7]. Furthermore, it has been documented that reduced antimicrobial pressure favors bacteria with single mutations to dominate, whereas continued antimicrobial pressure or new antimicrobial use favors bacteria with additional fluoroquinolone resistance [19].

There are multiple mechanisms of quinolone resistance and a common mechanism is mutations in the quinolone resistance determining region of target genes [20]. A single mutation in *gyrA* is known to cause resistance to nalidixic acid. However, resistance to fluoroquinolones such as ciprofloxacin is due to multiple mutations in *gyrA* and *parC*, and/or other mechanisms such as plasmid mediated quinolone resistance [21]. The *qepA* gene encodes efflux pumps that confer resistance to hydrophilic quinolones such as ciprofloxacin but not to hydrophobic quinolones such as nalidixic acid. Also, the *aac(6′)-Ib-cr* gene encodes an enzyme that inactivates fluoroquinolones such as ciprofloxacin [21].

Several risk factors have been associated with shedding quinolone resistant *E. coli* in pre-weaned dairy calves such as age less than 18 days, and recent fluoroquinolone use in a herd [22]. Another study reported quinolone resistance was most prevalent in 36 – 65d old calves [7]. Fluoroquinolone treatment is known to suppress *Enterobacteriaceae*, but this is followed some days or weeks later by increased prevalence in quinolone resistant *Enterobacteriaceae* [23]. Another explanation for quinolone resistance could be that quinolone resistant *E. coli* are common in the feces of pre-weaned dairy calves and the farm environment, hence, calves can acquire exogenous strains from the farm environment [24].

Reduced sulfonamide use was associated with decline in sulfonamide resistance and declining tetracycline resistance could be attributed to discontinued tetracycline use. Ampicillin was consistently used and ampicillin resistance remained consistent during the study period.

Aminoglycosides were not used in calves on this farm until late November when spectinomycin was introduced and used in a few calves. However, we detected high resistance to kanamycin and streptomycin and moderate resistance to gentamicin. Also, there was high chloramphenicol resistance despite lack of chloramphenicol use and low florfenicol use. Chloramphenicol and aminoglycoside resistance could be explained by co-selection [6]. Multidrug resistance could be due to presence of integrons and selection pressure from other antimicrobials. Class 1, 2 and 3 integrons play an important role in gene mobilization in *E. coli* and carry multiple resistance gene cassettes [25, 26]. In *E. coli*, integrons are often plasmid mediated and antimicrobial selection pressure is required for their acquisition and maintenance [27]. The persistence of chloramphenicol resistance has been attributed to chloramphenicol resistance genes on mobile genetic elements and efficient distribution via horizontal gene transfer [28].

Moderate ceftiofur resistance despite low ceftiofur use could be ascribed to selection pressure from ceftiofur use and co-selection. Ceftiofur use was formerly known to select for *E. coli* that carry *bla*_CMY-2_ genes on plasmids and confer resistance to ceftiofur [29, 30]. More recent studies indicate ceftiofur use selects for *E. coli* with *bla*_CTX-M_ genes encoded on plasmids and confer resistance to third generation cephalosporins such as ceftriaxone or cefotaxime [7, 31–33].

Though this farm had treatment protocols associated with syndromic disease (diarrhea, respiratory disease, and dehydration) we do not know the actual indications for the decisions of when a calf was sick and what the dosage of antimicrobials administered. Calf-treaters identified “sick calves” by daily observation of feeding behavior, attitude, and clinical signs such as diarrhea and cough, but a related study found that calf care workers identified “sick calves” based on their belief systems and made treatment decisions according to either their own beliefs of efficacy or followed the goals of the farm as manifested by treatment protocols [34]. While across the entire study period treatment decisions were made based on treater beliefs of the presence of disease, during the pre-antimicrobial policy change treaters chose the antimicrobials and mixed them based on their beliefs of efficacy. This practice was limited in subsequent time periods by the farm ownership and their veterinarian. Antimicrobial resistance testing was not performed for macrolides and this precluded evaluation of correlations between tylosin and tulathromycin use and macrolide resistance.

## Conclusions

This study documented antimicrobial use in pre-weaned calves and investigated the effects of changes implemented to reduce antimicrobial use on resistance. Nearly all calves received antimicrobials by weaning and antimicrobial use was more intense in younger calves (1–28d) compared to older calves. About 85% of the isolates were resistant to at least 3 classes of antimicrobials. Extensive resistance including resistance to fluoroquinolones and third generation cephalosporin (ceftiofur) was mostly observed in younger calves and calves that received multiple treatments. The observed resistance was most likely related to frequent antimicrobial use, the practice of combined antimicrobial therapy, and multiple treatments. Some of the observed resistance could be attributed to co-selection. Overall, the period before antimicrobial use policy change was implemented was associated with extensive resistance, whereas the periods thereafter, levels of resistance to most antimicrobials declined over time (except ampicillin, ciprofloxacin, ceftiofur). The most resistant class was mostly observed in 15 – 28d old calves after the policy change and this finding could be explained by continued use of ampicillin and enrofloxacin in this age group. Our results emphasize the importance of a continuum of record-keeping, validation of records, feedback of data, and active outreach and education as a cornerstone of antimicrobial stewardship on farms. In this study, farm management in response to feedback chose to adopt and implement new antimicrobial use policies that affected on farm resistance dynamics and reduced overall resistance.

## Methods

### Farm setting

This study was conducted on a single dairy farm in Washington State from 3 May 2016 to 30 January 2017. The farm housed 1200–1500 pre-weaned Holstein calves at a time. At parturition, calves were separated from the dam and fed 3.8 L of colostrum. Subsequently, calves were housed individually and for the first 21 days fed 1.9 L of bulk tank milk twice per day with approximately 15 g of bovine serum supplement (Gammulin, APC Inc., Anikeny, IA). At day 22 until weaning, calves were switched to receiving 1.9 L twice per day of a 22% protein and 20% fat calf milk replacer (Calva Products LLC, Acampo, CA) supplemented with approximately 5 g of oxytetracycline. Calves had access to ad-libitum water and grain supplement throughout the pre-weaning period and were weaned at approximately 60 days of age. The farm had treatment protocols associated with their routine observed syndromic diseases and antimicrobials associated with the protocols were available to on-farm personnel.

This study is part of a research project which monitored peri-parturient management practices that impact calf health and antimicrobial treatment. The hypothesis of the study was: routinely providing information to management on colostrum hygiene, passive transfer status, and calving events would influence the quality and consistency of peri-parturient management and focus health and treatment decisions on high risk calves (calves receiving suboptimal colostrum, calves with low total serum protein values, and calves involved with difficult calving events). On a weekly basis, study investigators hand delivered reports to management and discussed the results. The reports for each calf related its calving events, colostrum management, and colostrum quality to that calf’s health and treatment history. The reports were also sent to the consulting veterinarians and study investigators met with them on a monthly basis.

### Enrollment, health assessments, and antimicrobial treatment data

All calves born between 3 May and 25 July 2016 were enrolled into the first phase of the study and followed until weaning. At enrollment, calves were assigned to a location, birth date noted, and a blood sample collected to determine total serum protein. Calves were observed daily by calf-treaters and calves that were assessed to be sick were treated using a strategy designed by the consulting farm veterinarian, but the strategy could be amended by treaters based on their judgement. Treatment information was posted on calf hutches, from which research staff recorded, on a daily basis, drug name, and treatment date. These data were collected daily until calves were weaned. Data was entered into Excel® spreadsheets (Microsoft, Redmond WA) for storage and subsequent analysis.

The antimicrobial use policy in the first phase of the project was designed to address three syndromic “diseases”: diarrhea, septic pneumonia, and pneumonia observed in calves > 16 days of age. On farm calf care workers had available for use sulfa (oral), ampicillin (parenteral), and penicillin (parenteral) for calves observed with diarrhea. For septic pneumonia enrofloxacin (parenteral), sulfa (oral), and tulathromyicin (parenteral) were available to workers. For pneumonia in older calves tylosin (parenteral), sulfa (oral), penicillin (parenteral), tilmycosin (parenteral), and florfenicol (parenteral) were available to workers. The process for treatment decisions and choice of antibiotics to administer was solely determined by treaters. The criteria for deciding to treat, particularly for calves < 14 days of age were singly or a combination of observed diarrhea, inappetance, depression, and belief that a calf was at risk for being unhealthy. At the end of July, based on feedback information, the farm changed antimicrobial use policies and implemented them at the end of August. This was intended to eliminate the administration of antimicrobial combinations as well as reduce the number and types of antimicrobials available to treat pre-weaned calves and ultimately reduce the volume of antimicrobials given to calves. The policy removed penicillin and tylosin as treatment options and explicitly reserved enrofloxacin to be used only in calves > 16 days of age. The policy eliminated combination therapies. Within these restrictions, a treatment decision was still determined by workers.

With the new policies, it provided an opportunity to study their effects on resistance. We therefore continued to collect daily antimicrobial use data from all calves born between 26 July 2016 and 30 January 2017 with weekly or biweekly farm visits. Weaning dates for these calves were not recorded but assumed to follow the farm policy of weaning at 60 days of age. For analyses, calves were grouped into 5 age categories (1 – 14d, 15 – 28d, 29 – 42d, 43 – 56d, and 57 – 80d) to account for differences in disease pressure and antimicrobial use with age.

The study had three-time intervals for comparison: prior to the policy change (May – August 2016), during the policy change when pre-weaned calves were a mix of treatment policies (September – mid November 2016), and after the policy change (mid November 2016 – January 2017).

### Antimicrobial resistance data

#### Fecal sample collection

A cross-sectional sampling strategy was used to collect fecal samples from calves to isolate commensal *E. coli*. Baseline fecal samples were collected in June 2016 before antimicrobial use policy changes, and subsequent samples were collected bi-weekly from August 2016 – January 2017. A total of up to 11 fecal samples (1 sample per one-week age category) was collected from 1 to 11 weeks old calves every sampling visit. Calves were housed in individual hutches located in rows according to age and the first calf was sampled within one-week age intervals that had fresh fecal droppings. Approximately 5 g of feces was scooped from the hutch floor using a sterile wooden tongue depressor, put in a sterile plastic bag, placed in a cool-box and transported to the laboratory. Samples were placed in a refrigerator and processed within 24–48 h of collection.

#### Sample processing and *E. coli* isolation

1 g of feces was added to 9 mL of sterile normal saline and a 1:10 dilution of 10^− 1^ – 10^− 3^ series was made. Then 100 μL of each dilution was plated onto MacConkey agar plates using the spread plate method with sterile beads and incubated at 37 °C for about 18 h. *Staphylococcus aureus* ATCC 25923 and *E. coli* ATCC 25922 were used as negative and positive controls respectively. A total of 8 lactose positive isolates were picked from the MacConkey plates that had well isolated colonies, streaked for isolation onto Columbia blood agar plates and incubated at 37 °C for about 18 h. Thereafter, oxidase test and Kovacs indole test were performed to identify *E. coli* [35]. The first 4 lactose-positive, oxidase-negative, and indole-positive isolates were picked and banked for further analysis. *E. coli* ATCC 25922 and *S.* Typhimurium laboratory strain from University of California, Davis (lactose-negative, oxidase-negative, and indole-negative) were used as positive and negative controls respectively.

#### Antimicrobial resistance testing

Each isolate was tested for susceptibility to 12 antimicrobials: amikacin, ampicillin, ceftiofur, chloramphenicol, ciprofloxacin, gentamicin, kanamycin, nalidixic acid, streptomycin, sulfisoxazole, tetracycline, and trimethoprim/sulfamethoxazole [36] using a 96-well replicate microplate agar assay. Each 96-well microplate contained 72 test isolates in columns 1–3, 5–7, 9–11 and 16 blank wells (columns 4 and 8). Column 12 contained controls: *E. coli* ATCC 25922, *Pseudomonas aeruginosa* ATCC 27853, *S.* Typhimurium ATCC 29945, *S. aureus* ATCC 25923; and *S.* Newport S13990 and *S.* Typhimurium S8740 from *Salmonella* bank, Washington State University. A 96-well microplate replicator (Boekel Scientific, Feasterville, PA) was used to stamp the samples and controls from a 96-well microplate onto 3 Mueller-Hinton agar plates. The first plate had no antimicrobial and acted as a positive control for viability. The second plate had the low-end concentration for intermediate resistance, and the third plate had the low-end concentration for resistance as defined by Clinical Laboratory Standards Institute breakpoints when available (Additional file 1: Table S3). The Mueller-Hinton agar plates were incubated at 37 °C for 24 h and evaluated for growth. Bacterial growth was coded 1 and no growth was coded 0. The patterns 100, 110, and 111 were interpreted as susceptible, intermediate, and resistant respectively. Isolates with unusual patterns were retested using the disc diffusion assay [37].

### Data analysis

#### Sample size

The focus of sampling was to define age-level antimicrobial resistance in preweaneed dairy calves for the three-time periods included in the study (before, during, and after the antimicrobial use policy change) and assess relative changes in resistance between the time periods. Our assumptions for sample size were a starting high-level resistance prevalence of 50% in the youngest age group (before policy change) and a reduction of at least 30% in the ending resistance prevalence in that same age group (after policy change). The unit of interest was resistance profiles of fecal *Escherichia coli*. Based on a Type I error of 0.05 and a Type II error of 0.2 the number of isolates to assess per age group was 38 isolates (R Project for Statistical Computing Version 3.4, Base Package, Power.Prop.Test). For the analysis, we grouped calves into two-week intervals that resulted in 4 age risk groups and a total of 152 isolates per time period or 456 isolates for the study. Based on collecting 4 isolates per calf we needed to enroll at minimum 114 calves.

#### Analysis of antimicrobial use data

All data was stored in Excel® spreadsheets and analyzed using R version 3.4.0. A summary of the antimicrobials used and the percentage of calves treated with each antimicrobial was calculated and the age at which calves were treated was determined. The cumulative number of antimicrobial treatments given to a calf from birth to weaning was calculated, and the combination of antimicrobials used determined. Treatment intensity was defined as the number of calves treated with a particular antimicrobial per day per 100 calves of similar age (within two-week interval) [6, 10]. To assess trends, treatment intensity was stratified by age group and plotted over time and LOWESS function (locally weighted regression analysis) was used to fit a smoothing line to data [38].

#### Analysis of antimicrobial resistance data

Intermediate resistance and resistant isolates were reclassified as non-susceptible and each isolate was categorized into a single pattern of susceptible or non-susceptible to the 12 antimicrobials tested. The overall percentage of isolates that were non-susceptible to each antimicrobial was determined. The proportion of isolates resistant to each antimicrobial over time was plotted and trends assessed by simple linear regression analysis.

Latent class analysis (LCA) was performed using R package poLCA [39] to identify unique classes of isolates with shared resistance patterns. LCA is a statistical method that uses observed categorical responses to identify underlying latent or “unobserved” groups of individuals or objects that share certain characteristics [40]. This approach has been used to identify antimicrobial resistance structure [41].

To determine how changes in antimicrobial use affected resistance over time, we performed multinomial logistic regression analysis with LCA resistant class as the dependent variable using both univariable and multivariable models. The exposure variables modeled commonly used antimicrobials and we included age group as a potential confounder and evaluated whether sampling period was an effect modifier. These analyses were performed using R package nnet [42]. We performed cross tabulations of variables to determine the number of isolates in different cells and for cells with zero count, one count was added and we calculated odds ratio and standard error as previously described [43].

## Additional file

Additional file 1: **Table S1.** Antimicrobial combination treatments given to pre-weaned calves (*n* = 4301) on a dairy farm. **Table S2.** Criteria for latent class analysis model selection based on resistance to 11 antimicrobials. **Table S3.** Minimum inhibitory concentration (μg/ml) of antimicrobials used for agar dilution assay. (XLSX 14 kb)

## Data Availability

The datasets generated and analyzed during the current study are not publicly available due to privacy issues associated with agreements made with individuals and entities involved in the research but are available from the corresponding author on reasonable request.

## Abbreviations

enro: enrofloxacin
pen: penicillin
sul: sulfonamide
Tx: Treatment
